# Peptide Modified ZnO Nanoparticles as Gas Sensors Array for Volatile Organic Compounds (VOCs)

**DOI:** 10.3389/fchem.2018.00105

**Published:** 2018-04-16

**Authors:** Marcello Mascini, Sara Gaggiotti, Flavio Della Pelle, Corrado Di Natale, Sinazo Qakala, Emmanuel Iwuoha, Paola Pittia, Dario Compagnone

**Affiliations:** ^1^Faculty of Bioscience and Technology for Food, Agriculture and Environment, University of Teramo, Teramo, Italy; ^2^Department of Electronic Engineering, University of Roma Tor Vergata, Rome, Italy; ^3^Sensor Lab, Department of Chemistry, University of the Western Cape, Bellville, South Africa

**Keywords:** ZnO nanoparticles, peptides, virtual docking, quartz crystal microbalances, gas sensor array

## Abstract

In this work a peptide based gas sensor array based of ZnO nanoparticles (ZnONPs) has been realized. Four different pentapeptides molecularly modeled for alcohols and esters having cysteine as a common spacer have been immobilized onto ZnONPs. ZnONPs have been morphologically and spectroscopically characterized. Modified nanoparticles have been then deposited onto quartz crystal microbalances (QCMs) and used as gas sensors with nitrogen as carrier gas. Analysis of the pure compounds modeled demonstrated a nice fitting of modeling with real data. The peptide based ZnONPs had very low sensitivity to water, compared to previously studied AuNPs peptide based gas sensors allowing the use of the array on samples with high water content. Real samples of fruit juices have been assayed; stability of the signal, good repeatability, and discrimination ability of the array was achieved.

## Introduction

In the last years, gas sensor arrays, often reported as electronic noses, have proved to be very useful tools for the analysis of foods and flavors. These tools can be applied in quality control (to control system of production, shelf-life monitoring, evaluation of the food freshness) of the product or in the study of the volatility of aromas in a wide range of food as cheese, wine, coffee, tomato, meat etc (Ampuero and Bosset, 2003; Loutfi et al., 2015). The use of gas sensor arrays in the food industry represent an opportunity considering their high sensitivity, high correlation with conventional sensory evaluation, short analysis times, low cost, non-invasive measurement, and potentiality of automation (Duran and Marcato, 2013; Vanderroost et al., 2014).

One of the simplest configurations in gas sensing is represented by quartz crystal microbalances (QCMs). These sensors have the potential for sensitive and selective target gas detection due to the ability to measure mass change, on the surface of the sensor itself, after interaction between the sensitive film and the gas (Tokura et al., 2017). The sensing surface of QCMs can be easily modified with organic compounds taking advantage of the measurement run at room temperature. Considering organic molecules as binding elements, some attempts to use proteins has been made recenty with odorant binding proteins on QCMs (Sankaran et al., 2011a,b). However, either purification or production, via genetic engineering, of a pattern of odorant binding proteins for the construction of different gas sensor arrays is costly and time consuming. The use of short peptide sequences as binding elements in the gaseous phase is particularly interesting as the peptides can easily be synthesized and designed using virtual screening to bind molecular targets. It has been recently reported that peptides exhibit a high ability to bind volatile compounds, and that main factor influencing the bond is the chemical nature of the volatile compound (Martínez-Arellano et al., 2016). The feasibility of the use of peptides onto QCMs has been already demonstrated using the glutathione tripeptide and its amino acid components to develop an array of QCM-based gas sensors (Compagnone et al., 2013). A virtual screening approach has been later developed and validated testing the response of the QCM sensors for the detection of some VOCs (Pizzoni et al., 2014). Peptides selected via molecular modeling were used different times as affinity binders in analytical detection systems (Mascini et al., 2005, 2013; Baggiani et al., 2013; Heurich et al., 2013). Peptides rationally designed have been immobilized onto piezoelectric transducers working either in liquid (Mascini et al., 2006) or in gas (Mascini et al., 2017). These short peptides have been proved to give satisfactory results in the assay of different food commodities from olive oil (Del Carlo et al., 2014) to chocolate and candies (Compagnone et al., 2015; Pizzoni et al., 2015). The ability of peptide-based gas sensors to discriminate among off-flavored and regular samples of dark, white, and milk chocolate or among candies prepared with a natural or synthetic strawberry flavor was higher than classical phorphyrin based QCMs array. In the latter works, in order to improve sensitivity and the amount of the ligand onto each sensor, the peptides have been previously immobilized onto gold nanoparticles (AuNPs) using as spacer a cysteinyl-end for each of the peptides. AuNPs represented the first choice as nanomaterial since have been extensively studied for their many different proprieties and ability to be functionalized (Saha et al., 2012; Yue et al., 2016; Della Pelle and Compagnone, 2018).

In this work, we exploit the characteristics of ZnO nanoparticles to act as carrier for the peptide binding (Vallee et al., 2010) units of a gas sensor array. ZnO, as other metal oxides has an iso-electric point in alkaline range. This is a clear advantage for non-covalent immobilization of enzymes as most of the proteins operating in physiological range have isoelectric point in acidic range. Thus, immobilization is obtained principally through electrostatic interaction. As example, glucose and cholesterol enzyme electrodes have been realized with this approach (Ahmad et al., 2009; Ren et al., 2009), and different biosensors realized using ZnO have been very well-reviewed in a recent work (Arya et al., 2012).

Here, peptide-ZnO nanoparticles have been characterized and used as gas sensing array. Four different peptides were tested as gas sensing elements. The peptides were selected from molecular modeling in order to build an array able to discriminate among alcohols and esters. Principal component analysis of the eight different alcohols and esters tested clearly indicate discrimination among the two classes. Moreover, differently from peptide-AuNPs, signal of the peptides ZnONPs was not influenced by water content, opening the possibility to work with samples with high content of water. Assay of different fruit juices was reported in this respect.

## Materials and methods

### Virtual docking

A previously described docking procedure, reported in details in other works (Perez et al., 2013; Pizzoni et al., 2014), was applied to calculate the binding score between peptides and volatile compounds. Briefly, virtual binding preparation and processing was carried out using OpenEye Scientific Software package under academic license. Optimization of molecular geometries was achieved using SZYBKI with default parameterization (SZYBKI, version 1.5.7)^1^ Ten conformers per peptide and a maximum of 200 conformers for each volatile compound were generated by means of OMEGA (Hawkins et al., 2010; Hawkins and Nicholls, 2012; OMEGA, version 2.4.6)^2^ Multi-conformer rigid body docking was carried out using OEDocking 3.0.0, also with default parameters (OEDocking, version 3.0.0)^3^ The entire process was automated using AutoIT V3, a freeware BASIC-like scripting language. Physicochemical properties were calculated by using VIDA version 4.1.1 (VIDA, version 4.1.1)^4^ and an online tool for calculating peptide properties (http://www.innovagen.se/custom-peptide-synthesis/peptide-property-calculator/peptideproperty-calculator.asp).

### ZnONPs characterization and functionalization

All reagents and the eight volatile compounds used were purchased from Sigma-Aldrich (Italy). The eight volatile compounds were of analytical grade. The four peptides (IHRIC, LAWHC, TGKFC, and WHVSC) used for ZnO functionalization were purchased from Espikem (Italy, purity > 85%). ZnONPs were synthesized following the procedure used in another work (Zak et al., 2011): 0.5 M zinc acetate ethanolic solution was added with 1:1 molar ratio triethanolamine and stirred for 1 h at 60°C. The solution was then kept at room temperature 1 h and at 150°C for 18 h. The formed precipitate was dispersed in ethanol cleaned three times by centrifugation and then dried in an oven at 60°C overnight.

The nanoparticles were characterized by high resolution transmission electron microscopy (HRTEM) using (TEM, S-2400 N, HITACHI, Japan). The samples for HRTEM characterization were prepared by placing a drop of the dilute sample solution on a carbon-coated copper grid and dried at room temperature before measurements. High resolution scanning electron microscopy (HRSEM) images were recorded using a Hitachi S3000N scanning electron microscope at an acceleration voltage of 20 kV and a magnification of 100000.

ZnONPs functionalization was achieved by adding an aliquot of 100 μL of a 10^−3^ M aqueous solution of coating peptide to 900 μL of ethanol/H_2_O 9:1 v/v suspension of ZnONPs. The ZnONPs suspensions were kept overnight at room temperature and then stored at 5°C.

Fourier Transform Infrared (FTIR) spectra of ZnONPs and ZnONPs-Peptide were recorded in the range 4,000–500 cm^−1^ using a Perkin Elmer model Spectrum 100 series. The ultraviolet-visible (UV-Vis) spectra were recorded on a Nicolet Evolution 100 in the range 200–800 nm.

### QCM sensors assembly and measurement setup

Twenty MHz QCM sensors, were from KVG GmbH (Neckarbischofsheim, Germany). The QCM sensors modification was achieved by drop casting 5 μL of the ZnONPs-peptide suspension on each side of the crystal and let to dry for few minutes. Before the first use, the QCM were then completely dried under N_2_ at a flow rate of 2 L/h. QCMs were stored at room temperature in the dark when not in use.

The piezoelectric measurements were carried out using a TEN 2009 E-nose (Sensor group, University of Rome Tor Vergata, Italy). Carrier gas was N_2_ used at a flow rate of 2 L/h.

Measurements of the alcohols and aldehydes were done as described in other work (Pizzoni et al., 2014). Analysis of the real samples was run using 500 μL aliquots of juice fruit in glass lab bottles (100 mL) heated at 24°C. The sample was kept for 5 min at 24°C. This range of time was selected as the optimum time required to reach a steady state by the juice fruit volatile compounds in the headspace sample volume. The N_2_ enriched with the volatiles present in the vapor phase of the real sample was then assayed by the E-nose for 15 min.

The stop-cocks were then opened to carry the head-space of the 100 mL glass bottle to the measuring sensor array chamber and the frequency shift (ΔF), taken as analytical signal, was recorded. After each measurement, a complete recovery of the signal was achieved under N_2_ flow in about 400 s. The piezoelectric sensorgram was similar for all peptides and volatile compounds, showing a rapid decrease of the signal after the stop-cocks opening, followed by a slower raise up to the steady state. The ΔF, was recorded for all compounds after 600 s.

In order to highlight the affinity between volatile compounds and sensors, concentration effect was removed normalizing the volatile compound response of each peptide sensor with the following equation (Di Natale et al., 2003)

$$\begin{array}{l} \frac{\Delta Fi}{\sum_{i=1}^{n}\Delta Fi} \end{array}$$

where Δ*F* was the frequency shift of each sensor and *n* the number of sensors in the array, in this case four. Assuming valid the Sauerbrey law, such normalization is supposed to remove the effect of the concentration in the case of linearity of the sensor/signal volatile concentration.

### Multivariate analysis

Piezoelectric responses dataset was analyzed by the unsupervised multivariate technique principal component analysis (PCA) using MatLab R2011 (Mathworks, Natick, MA, USA) Row normalized data were autoscaled (zero mean and unitary variance) before analysis. PCA was applied to inspect the multivariate data structure by decomposing a data matrix of eight rows (the volatile compounds) and four columns (the peptides). PCA achieves this by computing the eigenvectors and eigenvalues of the covariance matrix of the dataset. PCA can be also used as a tool to reduce the dimensions of the dataset while retaining the major variation of the data. PCA has been used by many researchers for monitoring the changes in volatiles as recorded by e-nose (Baietto and Wilson, 2015).

## Results and discussion

### Virtual docking of peptides vs. volatile compounds

ZnO nanoparticles, having a high isoelectric point, provide a positive electrostatic surface suitable for interacting with negatively charged groups (Kumar and Chen, 2008; Dai et al., 2009). The peptides were immobilized by physical adsorption onto the ZnO nanoparticles taking advantage of cysteine terminal carboxyl group that in ethanol/H_2_O (90/10 v/v, pH~7) was negatively charged. Cysteine was also used as spacer to give orientation to the peptides binding. Peptides having aspartic or glutamic acid as residual groups were not used because it was not possible to predict the immobilization layout. As reported in Table 1 the peptides chosen in this work had positive or neutral net charge at pH 7 with in all cases a positive formal charge.

**Table 1:** Structural and physicochemical properties of the four peptides and eight volatile compounds used in this work.

	**Net charge**	**Isoelectric point**	**Formal charge**	**MW**	**LogP**	**PSA**	**Rot_B**	**Acc**	**Don**
Peptides	pH 7	pH							
IHRIC	1	8.87	2	643	−1.38	277	25	7	10
LAWHC	0	7.08	1	630	−0.10	228	20	7	7
TGKFC	0.9	8.54	1	556	−1.78	232	21	7	7
WHVSC	0	7.07	1	632	−1.66	250	20	7	9
**Volatile compound**	**Chemical class**	**Structure**	**N Confs**	**MW**	**LogP**	**PSA**	**Rot_B**	**Acc**	**Don**
1-butanol	Alcohols	Short chain	5	74	0.83	20	2	1	1
1-hexanol	Alcohols	Long chain	36	104	1.82	20	4	1	1
2-methyl-1-propanol	Alcohols	Short chain	2	74	0.59	20	1	1	1
Ethanol	Alcohols	Short chain	1	46	−0.16	20	0	1	1
Hex-3-en-1-ol	Alcohols	Alkene	18	100	0.75	20	3	1	1
Ethyl acetate	Esters	Short chain	3	88	0.54	26	2	1	0
Ethyl-methyl-2-butyrate	Esters	Long chain	49	130	1.82	26	4	1	0
Isopentyl acetate	Esters	Long chain	9	130	1.78	26	4	1	0

*N confs, number of conformers; MW, molecular weight; PSA, polar surface area; RB, rotatable bond; Acc, Lipinski acceptors; Don, Lipinski donors*.

The partition coefficient, logP, was lower than one resulting in negative logarithmic value for all peptides with slight hydrophobic tendency only for LAWHC. This peptide had also the smallest PSA (polar surface area) value reinforcing the trend of the logP. The volatile compounds tested having short or long alkyl chains were from two chemical classes; alcohols and esters. Only hex-3-en-1-ol contained a double bond and, as shown by the number of conformers, had high flexibility along with hexanol and ethyl-methyl-2-butyrate. All volatile compounds, except ethanol, had good affinity for hydrophobic phase with similar PSA.

The peptides-volatile compounds interactions cannot be correlated to a single physicochemical property, but to the contribution of each residue in the specific space occupied within the peptide. Therefore, the binding properties of the four peptides vs. the eight volatile compounds were tested considering the whole peptide as possible binding site for ligands. Molecular modeling study was addressed to reliably screen out compounds that did not fit in the binding site or that had grossly wrong electrostatic properties. In fact, the ability to distinguish between two compounds that both bind to the same active site is modest using this approach.

The percentage of the docking scores calculated over 10 conformers for each peptide are reported in Table 2, where higher values represent higher peptide–volatile compound affinity. All peptides had an overall trend in binding better alcohols than esters. Differences between chemical class average and single compounds of the same class were found, particularly for peptides TGKFC and WHVSC responses vs. hexen-3-en-1-ol. In the alcohols class, there was a clear different binding response between short and long alkyl chains with the lowest interaction for ethanol given by peptide LAWHC. This peptide was the only one having an esters binding score trend better than short alkyl chain alcohols. A relative good binding ability for esters was also exhibited by peptide TGKFC.

**Table 2:** Percentage of the binding score, representing the affinity of the peptides toward the volatile compounds used in experimental part.

	**IHRIC (%)**	**LAWHC (%)**	**TGKFC (%)**	**WHVSC (%)**
1-butanol	60	61	81	66
1-hexanol	64	89	85	82
2-methyl-1-propanol	61	63	79	68
Ethanol	60	54	68	61
Hexen-3-en-1-ol	67	79	100	90
Ethyl acetate	49	63	62	37
Ethyl-methyl-2-butyrate	30	68	51	28
Isopentyl acetate	33	60	48	33

*The binding score average of each peptide receptor was calculated over 10 peptide conformers, the coefficient of variation ranged between 5 and 15% in all cases*.

### ZnONPs-peptides characterization and functionalization

**Figure 1:**
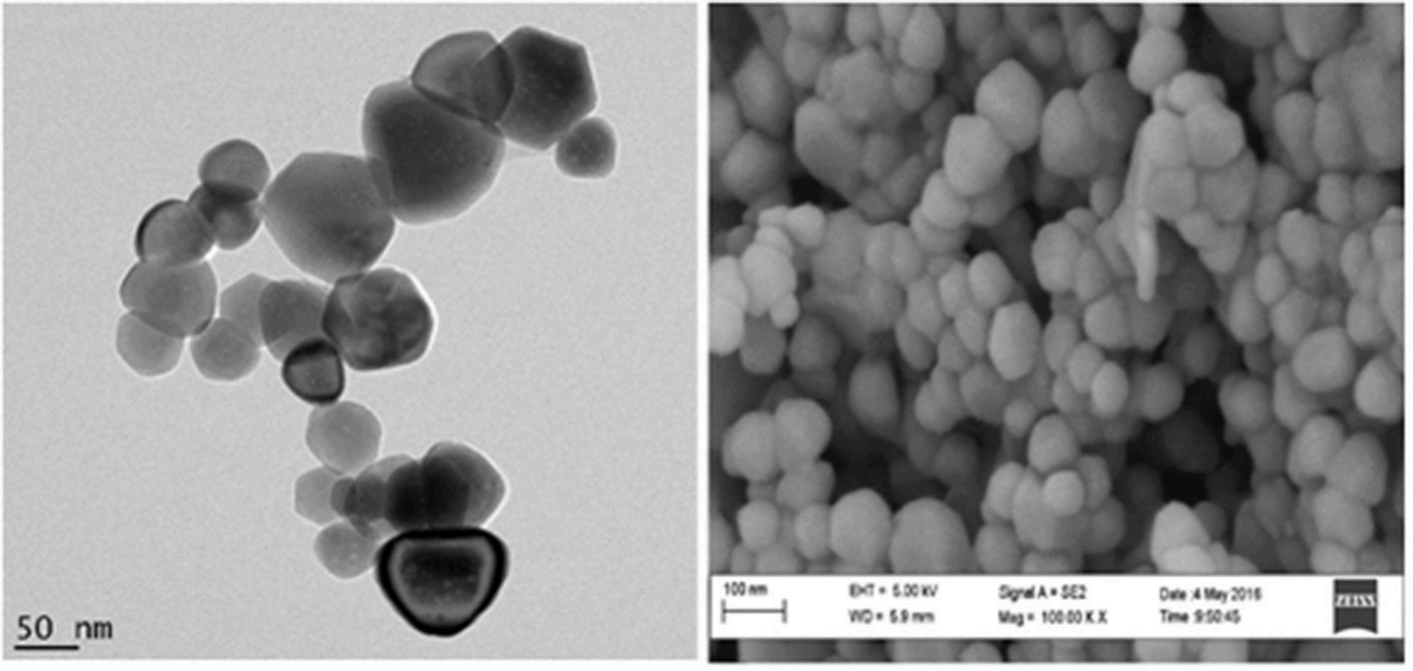
HRTEM and HRSEM (100000x magnification) images of the dried ZnONP powder redissolved in ethanolic solution.

The synthesized zinc oxide nanoparticles were characterized using transmission high resolution scanning electron microscopy (HRSEM) and transmission electron microscopy (TEM) to determine the size of the zinc oxide nanoparticles. Figure 1 reports the classical cubic shapes obtained with an average diameter lower than 100 nm. The SEM figure indicates a homogenous shape and size for ZnONPs. It also shows that the ZnONPs are well-dispersed in the powder form.

**Figure 2:**
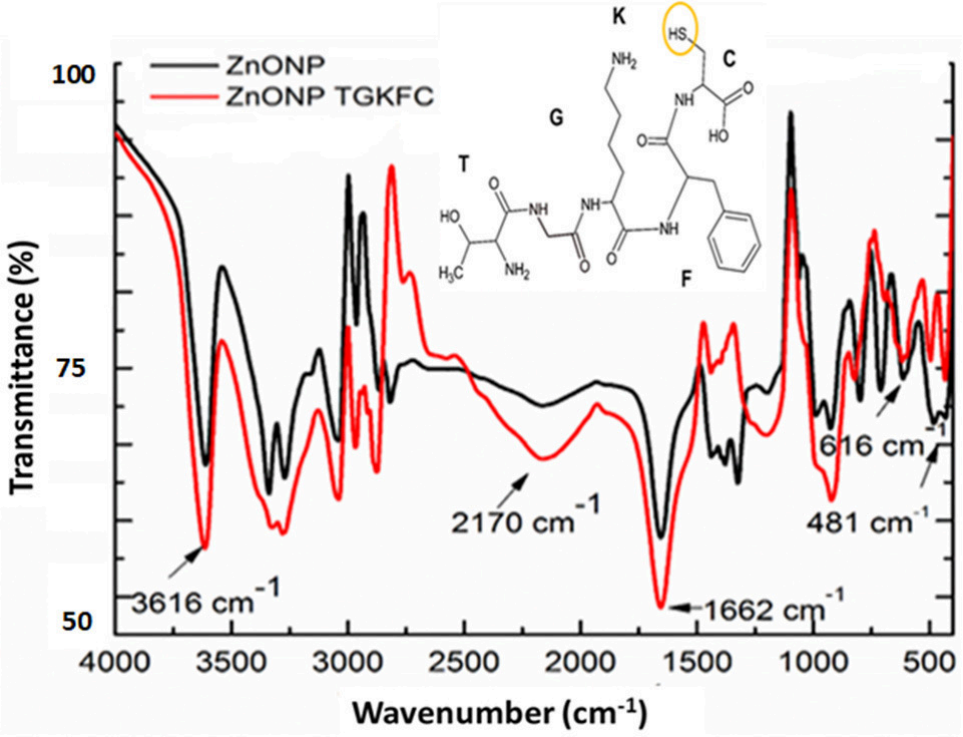
Fourier transform infrared spectra of ZnONPs-TGKFC. Black line ZnONPs alone; Red line the ZnONPs bound to TGKFC. The molecular structure of the peptide was also reported highlighting the functional group involved in the stretching frequencies.

UV/Vis absorption spectra of ethanol/ water (9:1) suspensions of ZnONPs exhibited a distinct sharp absorption band at 370 nm as reported in the literature (Zak et al., 2011); modification with the peptides did not result in any significant change of the UV spectra. A clear proof of the immobilization of peptides onto ZnONPs was obtained using FTIR spectroscopy. Changes in peak intensity and shape upon binding was observed together with the appearance of new peaks as reported in Figure 2 for the spectra of ZnONPs vs. the TGKFC peptide modified ZnONPs.

As shown in Figure 2, the band appearing at 481 cm^−1^ was assigned to the zinc oxide (Jurablu et al., 2015). The FTIR stretching frequencies observed in the spectra of unmodified ZnONP were either due to the residual of the acetate ions and ethanol solvent. These should result from the stretching frequencies of the functional groups C = O occurring in the 1,670–1,820 cm^−1^ region, C-O in the 1,050–1,150 cm^−1^ region, and C-H observed at 1,000–1,300 cm^−1^, with strong intensities.

The appearance of the band at 1,663 cm^−1^, can be assigned to the stretch vibration of C = O with strong intensity associated with air from the atmosphere (Djelloul et al., 2010; Dutta and Ganguly, 2012; Kumar and Rani, 2013). The band at 3,616 cm^−1^, is due to the stretch vibration of O-H and could be associated with the ethanol from the diluted nanoparticles (Zak et al., 2011) (Dehaghi et al., 2014).

It is expected that the thiol group of the TGKFC can interact with zinc metal to form ZnONP-TGKFC complexes. The peptide should use the thiol SH group of the peptide sequence to bind to the surface of the ZnO nanoparticles. Infact, the thiol group has high binding affinity with the metal ions (Badia et al., 2000). Looking at the peptide structure, there is a presence of υ(O-H) at 3,612 cm^−1^, assigned to the stretch vibration of the O-H from the L-cyteine (C) carboxylic acid derivative of the peptide sequence. The υ(C = O) observed at 1,662 cm^−1^ with strong intensity, are stretching frequencies resulting from the peptide bond C = O functional groups between the amino acid residues overlapping with the C = O funtional groups stretching frequencies of the free-carboxylic group of the L-cysteine derivatives. The weak band at 3,200 cm^−1^ was due to the peptide bond tertiary amine υ(N-H) streching frequency. The medium band observed at 3,350 cm^−1^ was assigned to the υ(-NH_2_) primary amines of the threonine (T) and lysine (K) amino acid derivatives of the amino acid peptide sequence. Aromatic stretches; υ(C = C) of the aromatic benzene ring of the phenylalanine (F) amino acid peptide residue stretching frequencies were also observed at 1,420 cm^−1^ as multiple bands. Functionalizing the zinc oxide nanoparticles with the TGKFC peptide sequence also resulted in other unique bands formed at 2,818 cm^−1^ due to the C-H functional group alkyl chains of the multiple amino acid residues and the stretch vibration band at 1,339 cm^−1^ assigned to the stretch vibration at C-N functional groups. In this context it can be observed that there is an influence of the peptide as the stretching mode have been shifted to higher energy state.

### Piezoelectric array response

The cysteine spacer, with negative carboxyl terminal group used to immobilize each peptides onto positively charged ZnO nanoparticles, was supposed to preserve the three-dimensional shape of peptides with no or very little loss of affinity properties. QCM microbalance system frequency shifts (ΔF) were used in order to discriminate among the eight volatile compounds and to demonstrate the matching between the predictive ability of the virtual screening with real binding data. For that reason, pure volatile compounds were tested using nitrogen as carrier gas. To evaluate the sensors affinity with respect to the affinities of other sensors in the array it was necessary to remove the information about the concentration by dividing the signal of each sensor by the sum of the signals of all sensors in the array (Pizzoni et al., 2014; Mascini et al., 2017). Then, to emphasize the contribution that each sensor brings to the total array a column normalization was carried out The percentage of the normalized dataset response of each sensor is reported in Table 3.

**Table 3:** Percentage of the normalized frequency shift of the four peptide-modified sensors vs. the eight volatile compounds.

	**IHRIC (%)**	**LAWHC (%)**	**TGKFC (%)**	**WHVSC (%)**
1-butanol	11.8 ± 1.1	15.2 ± 1.3	11.3 ± 2.6	10.1 ± 2.2
1-hexanol	11.5 ± 2.8	13.7 ± 2.1	13.8 ± 3.6	13.0 ± 2.5
2-methyl-1-propanol	10.5 ± 0.9	17.2 ± 1.7	11.2 ± 2.5	10.3 ± 2.2
Ethanol	12.6 ± 2.4	8.3 ± 1.2	19.4 ± 3.6	17.5 ± 1.0
Hexen-3-en-1-ol	14.5 ± 0.4	5.4 ± 1.0	13.4 ± 3.1	18.9 ± 3.2
Ethyl acetate	12.0 ± 0.2	16.1 ± 0.9	11.4 ± 1.4	8.1 ± 0.6
Ethyl-methyl-2-butyrate	13.4 ± 0.9	13.1 ± 2.3	10.6 ± 2.6	9.6 ± 2.0
Isopentyl acetate	13.7 ± 0.3	11.1 ± 1.6	8.9 ± 1.9	12.5 ± 1.9

*The standard deviation was calculated using three measurements taken in 3 different days*.

The ZnO-IHRIC modified sensor had similar interactions with all the eight volatile compounds, this trend was also observed in modeling with the exception of long chain esters. The other three peptides exhibited a recognition pattern in binding the eight volatile compounds different from each other. The peptide LAWHC had strong interaction with long chain alcohols and esters but not with ethanol and surprisingly neither with hexen-3-en-1-ol, having the lowest interaction. This last behavior was not observed in modeling. With the exception of hexen-3-en-1-ol and isopentyl acetate, both peptides TGKFC and WHVSC had similar trend having better interaction with alcohols than esters, as also indicated by the modeled data. The fact that, experimental data did not fully overlap the simulated data can be attributed to the not well-defined orientation achieved because of the physical absorption of the peptides on ZnO nanoparticles.

**Figure 3:**
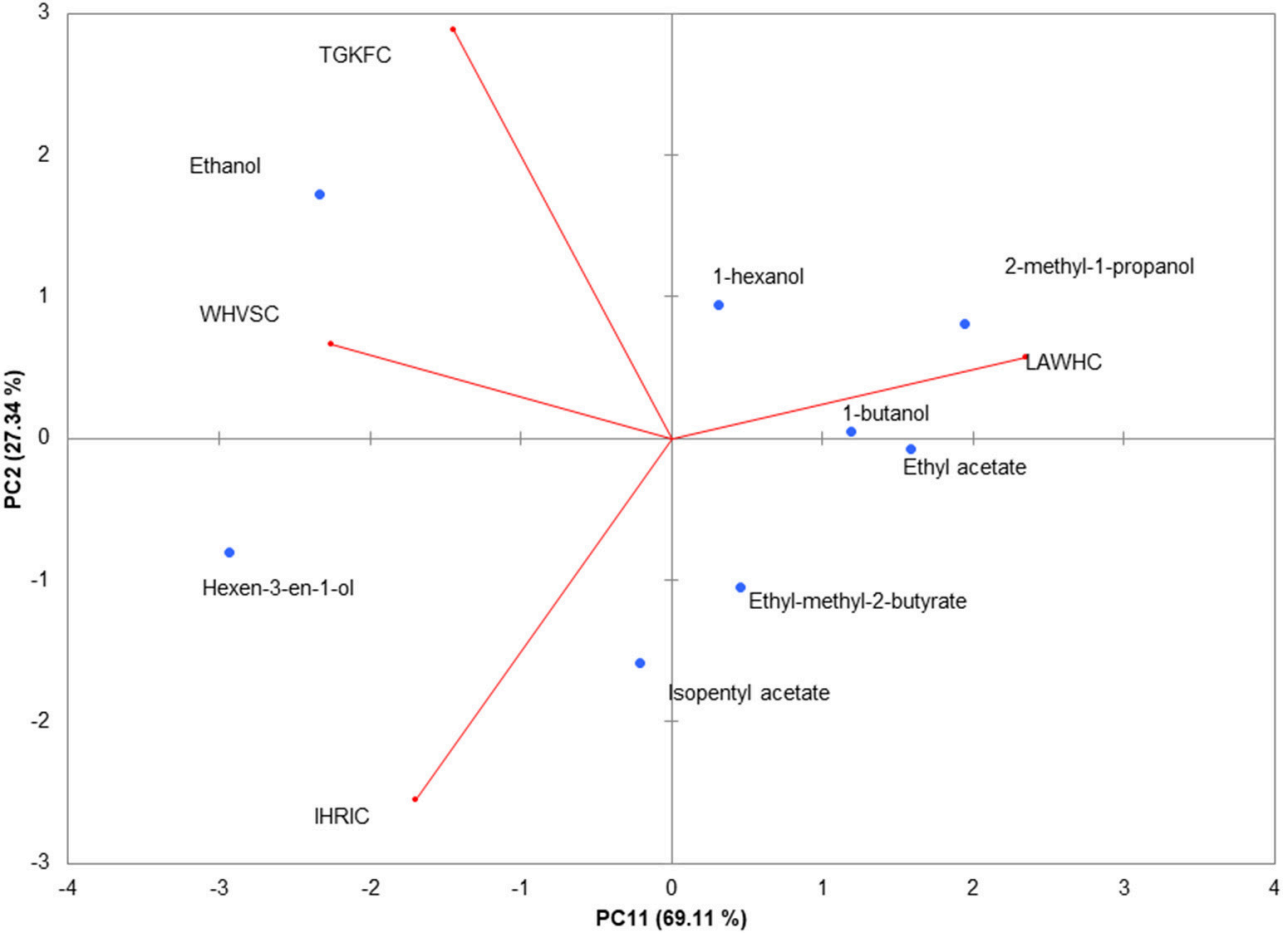
PCA of the piezoelectric response. The biplot (Score and loading) of the first two principal components showed 94.45% of the cumulative variance. Rows normalization were applied to the gas sensors array dataset. Data were autoscaled before PCA.

To better highlight the contribution of each sensor to the discrimination of the eight volatile compounds, the normalized data were analyzed making use of unsupervised PCA. The biplot of the first two principal components is shown in Figure 3. The first component represented 69.11% of the variance; the second 27.34% displaying together a cumulative variance of 96.45%. The behavior of volatile compounds can be appreciated looking at the scores points assuming that close distance in biplot plane is a measure of the similitude between samples. The changes of structural composition are well-captured by the PCA; in particular PC1 contributed to a clear separation between ethanol and hexen-3-en-1-ol from the others molecules. Alcohols, with the exception of hexen-3-en-1-ol, were clustered by the PC2 that scattered the esters on the fourth quadrant of PC1-PC2 plane.

The contribution of the peptides to the volatile compounds analysis can be evaluated considering the position of the loadings (the contribution of each variable to the principal components) with respect to the scores. Peptide LAWHC had a pattern recognition performance opposite to the other three peptides, contributing significantly to the separation of the esters and alcohols from ethanol and hexen-3-en-1-ol. This behavior fitted well with the simulation considering the hexen-3-en-1-ol affinity for peptides TGKFC and WHVSC. These two peptides together with IHRIC contributed to the separation of alcohols from esters on the PC2, confirming in part the trend obtained by virtual binding scores.

### Analysis in water and fruit juices

**Figure 4:**
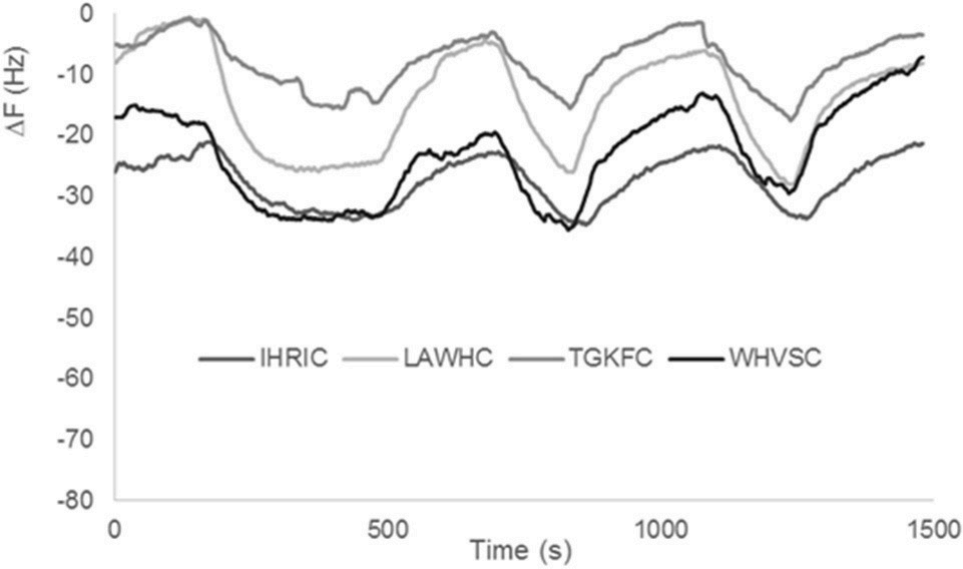
Frequency signals recorded with ZnONPs-peptides testing pure water.

Previous gas sensor arrays based on peptide modified AuNPs were used on samples with low content of water (i.e., candies, chocolate, olive oil). In fact, as already reported in the first realized peptide gas sensor arrays (Compagnone et al., 2013) pure water produced a drift of the signal. In following works, substituting citrate to borate in the production of AuNPs, drift from water was eliminated (Pizzoni et al., 2014, 2015; Compagnone et al., 2015; Mascini et al., 2017); however, high contents of water in mixture with other solvents, produced very high signals reducing the discriminating ability of the array and limited the use of the peptide based gas sensors. Peptide based ZnONPs did not interact to the same extent as AuNPs with water. Signals obtained with pure water for the four gas sensors are reported in Figure 4. It has to be noticed the low signal for all sensors. Stability of the signal was also evident as well as reproducibility, being the interday relative standard deviation (RSD) for all sensors tested below 10%. The small differences among the peptides can be attributed to a different degree of ZnO nanoparticles coverage that is difficult to assess accurately. The large difference between AuNPs and ZnONPs (response in the same conditions is hundreds of Hz for AuNPs) can be attributed either to the presence of higher charge density of Au^+3^ vs. Zn^+2^ onto the nanoparticles or to the rejection effect of oxygen in the highly polar ZnONPs.

**Figure 5:**
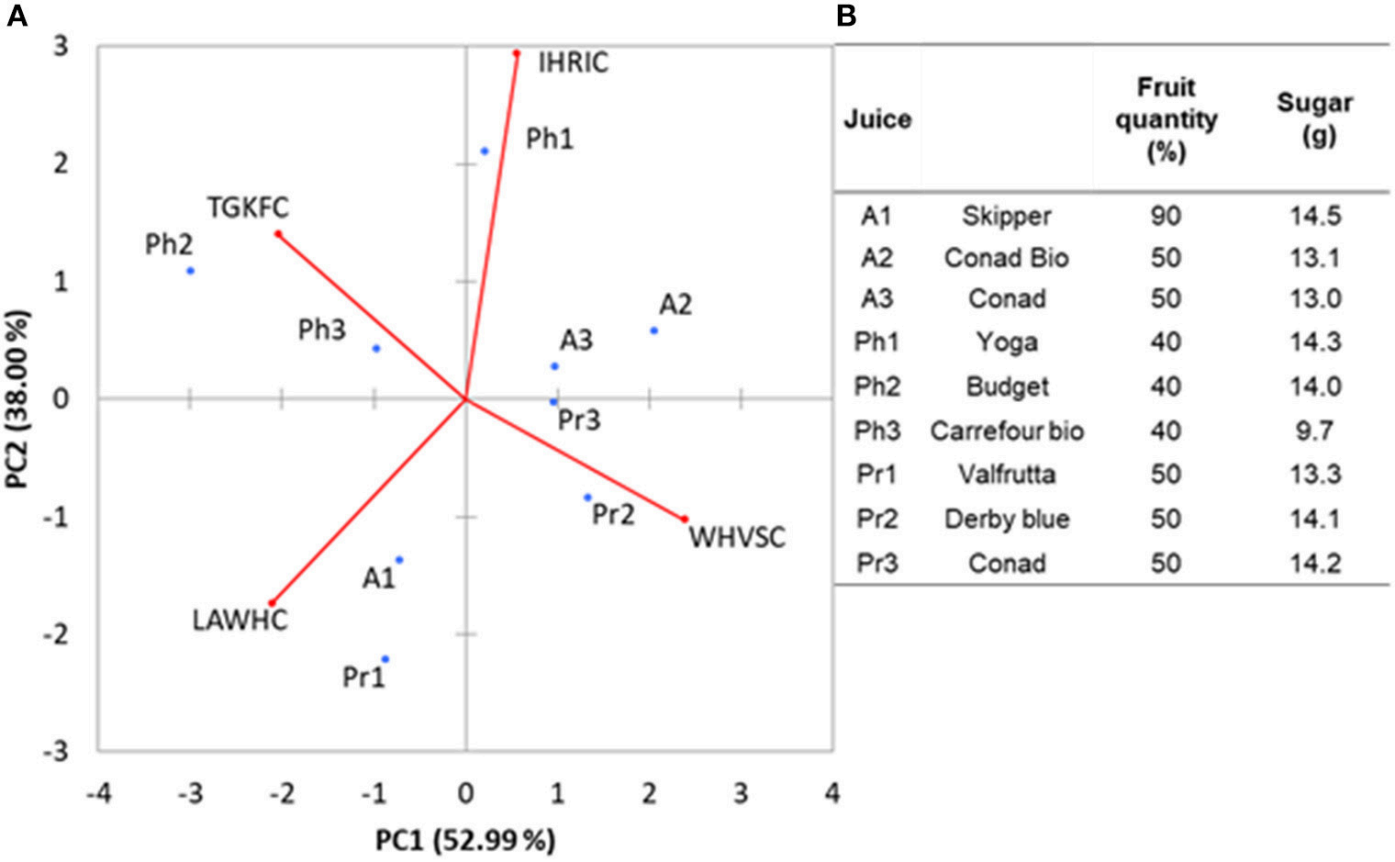
**(A)** The biplot values (sores and loadings) for fruit juices analysis along with the percentage **(B)** of fruit quantity and sugar concentration in the different fruit juices.

In order to demonstrate the ability of the array to carry out analysis on samples with high water content, nine different fruit juices bought at local markets were tested. E-noses based on metal-oxide gas sensors have been already successfully used for analysis of fruits (peaches) and fruit juices (apple) (Zhang et al., 2012; Wu et al., 2017). We selected this kind of samples because alcohols and esters are among the major components of the volatile fraction of juices. The aim was to demonstrate that the peptide based ZnONPs QCMs sensors can be used to classify this kind of food samples. The fruit juices were randomly tested in a period of time of 2 weeks. Interday RSDs were always below 20% for each of the samples. The PCA analysis, reported in Figure 5A, gave 91% of the total variance explained by the first two PCA. Loadings were well-distributed among the quadrants demonstrating that each of the peptide gas sensor contributed to the discrimination of the samples. Looking at the plot of the fruit juice scores, Figure 5B, differences about sugar content and the amount of fruit can be noticed. Apricot (A1 Skipper) has the highest content of fruit (%) and sugars (g) reported and is clearly separated by the other two Apricot samples (A2 Conad Bio and A3 Conad) having the same amounts of fruit and sugars. Pear fruit juices Pr2 and Pr3 (Derby Blue and Conad, respectively) have the same content of fruit and sugar and appear in the same quadrant while Pr1 (Valfrutta) contains lower sugar and is separated by the others in the PCA. Peach Fruit juices (Ph1 Yoga), (Ph2 Budget), (Ph3 Carrefour Bio) does not appear to be clearly discriminated by the different amount of sugars in the samples. This results demonstrated that the sensors can be used to study volatiles organic compounds of fruit juices since it is well-known that fruit content and amount of sugar influence the hedsoace pattern of these samples. Further studies including more peptide-based sensors and with homogenueous type of samples will be necessary to ascertain the performance vs. other type of e-noses.

## Conclusions

The work has demonstrated the feasibility of the construction of a peptide gas sensor array based on ZnONPs. Four different peptides, selected computationally for binding alcohols and esters have been immobilized onto ZnONPs, deposited onto QCMs and tested with the pure compounds. Experimental data fitted well with modeling. The gas sensor array was then challenged with water and with fruit juices. Data demonstrated that the peptide based ZnONPs gas sensors can be used profitably for high water content samples.

## Author contributions

MM run the molecular modeling and worked out comparison with real measurement. SG and FD prepared the sensors and run the measurements with the sensor array. CD run and interpreted multivariate data. SQ and EI run and commented charachterization of the nanomaterials. PP selected the volatiles and food samples. DC supervised the work and wrote the paper collecting the different contributions.

### Conflict of interest statement

The authors declare that the research was conducted in the absence of any commercial or financial relationships that could be construed as a potential conflict of interest. The handling Editor declared a past co-authorship with one of the author CD.

## Footnotes

^1^SZYBKI (version 1.5.7) *OpenEye Scientific Software*, Santa Fe, NM. Available online at: http://www.eyesopen.com

^2^OMEGA (version 2.4.6) *OpenEye Scientific Software*. Santa Fe, NM. Available online at: http://www.eyesopen.com

^3^OEDocking (version 3.0.0) *OpenEye Scientific Software*. Santa Fe, NM. Available online at http://www.eyesopen.com

^4^VIDA (version 4.1.1) *OpenEye Scientific Software*. Santa Fe, NM. Available online at: http://www.eyesopen.com
